# A new stegosaur (Dinosauria: Ornithischia) from the Middle Jurassic of Gansu Province, China

**DOI:** 10.1038/s41598-024-66280-x

**Published:** 2024-07-02

**Authors:** Li Ning, Susannah C. R. Maidment, Li Daqing, You Hailu, Peng Guangzhao

**Affiliations:** 1 School of Earth Sciences and Resources, China University of Geosciences (Beijing), Beijing, China; 2 Fossil Reptiles, Amphibians and Birds Section, Natural History Museum, London, UK; 3 Institute of Vertebrate Paleontology, Gansu Agricultural University, Lanzhou, Gansu China; 4 Key Laboratory of Vertebrate Evolution and Human Origins, Institute of Vertebrate Paleontology and Paleoanthropology, Chinese Academy of Sciences, Beijing, China; 5 College of Earth and Planetary Sciences, University of Chinese Academy of Sciences, Beijing, China; 6 Zigong Dinosaur Museum, Zigong, Sichuan China

**Keywords:** Dinosaur, Stegosaur, Middle Jurassic, Gansu, Palaeontology, Phylogenetics

## Abstract

Stegosaurs are a minor but iconic clade of ornithischian dinosaurs, yet due to a poor fossil record, their early evolution is poorly understood. Here, we describe a new stegosaur, *Baiyinosaurus baojiensis*, gen. et sp. nov. from the Middle Jurassic Wangjiashan Formation of the Pingchuan District, Baiyin City, Gansu Province, China. The frontal of *Baiyinosaurus* possesses a unique characteristic among Stegosauria: it is wider than long and contributes to both the medial and anterior margins of the supratemporal fenestra. The character combinations of dorsal vertebrae of *Baiyinosaurus* are also different to other stegosaurs: its neural arches are not greatly elongated, its parapophyses are well developed, and its neural spines are axially expanded in lateral. The features of the frontal and vertebrae of *Baiyinosaurus* are reminiscent of basally branching thyreophorans, indicating that *Baiyinosaurus* is transitional in morphology between early thyreophorans and early-diverging stegosaurs. Systematic analysis shows that *Baiyinosaurus* is an early-diverging stegosaur.

## Introduction

The stegosaurs are characterised by the possession of two parasagittal rows of hypertrophied dermal armour plates and/or spines extending from the neck to the end of the tail and have been found on all continents except for Antarctica and Australia^1^. The earliest stegosaurs are from the Middle Jurassic, they achieved a global distribution by the Late Jurassic and subsequently waned in diversity during the Early Cretaceous. Records of stegosaurs from the Middle Jurassic are rare, including only five taxa: *Loricatosaurus*^2^ from the U.K., *Isaberrysaura*^3^ from Argentina, *Adratiklit*^4^ from Morocco, and *Huayangosaurus*^5^ and *Bashanosaurus*^6^ from China.

Dinosaur fossils are abundant in Gansu Province. Since the first dinosaur was discovered in Gansu Province in 1930^7^, seventeen dinosaurian taxa have been discovered and named. Among these named dinosaurs, there are five sauropods including *Gobititan shenzhouensis* (Titanosauriformes)^8^, *Huanghetitan liujiaxiaensis* (Titanosauriformes)^9^, *Daxiatitan binglingi* (Titanosauriformes)^10^, *Qiaowanlong kangxii* (Brachiosauridae)^11^, and *Yongjinglong datangi* (Titanosauria)^12^; three theropods, including *Suzhousaurus megatherioides* (Therizinosauroidea)^13^, *Xiongguanlong baimoensis* (Tyrannosauroidea)^14^, and *Beishanlong grandis* (Ornithomimosauria)^15^, and nine ornithischians, including *Gongpoquanlong mazongshanensis* (Hadrosauroidea)^16^, *Archaeoceratops oshimai* (Ceratopsia)^17^, *Equijubus normani* (Hadrosauroidea)^18^, *Auroraceratops rugosus* (Ceratopsia)^19^, *Lanzhousaurus magnidens* (Iguanodontia)^20^, *Jintasaurus meniscus* (Hadrosauroidea)^21^, *Archaeoceratops yujingziensis* (Ceratopsia)^22^, *Xuwulong yueluni* (Hadrosauroidea)^23^, and *Taohelong jinchengensis* (Ankylosauria)^24^. Recently, astegosaurian specimen from the Lower Cretaceous Hekou Group of the Zhongpu area, Lanzhou-Minhe Basin, Gansu Province was reported, which is the first stegosaurian dinosaur from Gansu Province^25^. In 2016, Dr. Li Daqing and his team discovered some dinosaurs, including a stegosaur, several large-sized theropod and sauropod remains and one small theropod track site in Pinchuan District of Baiyin City, Gansu Province. Thereafter, these specimens were excavated and prepared by the Gansu Zhendan Dinosaur Culture Communication Co. Ltd., and they were preserved at Gansu Agricultural University. We herein describe the new stegosaurian dinosaur from the Middle Jurassic of Gansu Province.

## Results

### Geological setting

**Figure 1:**
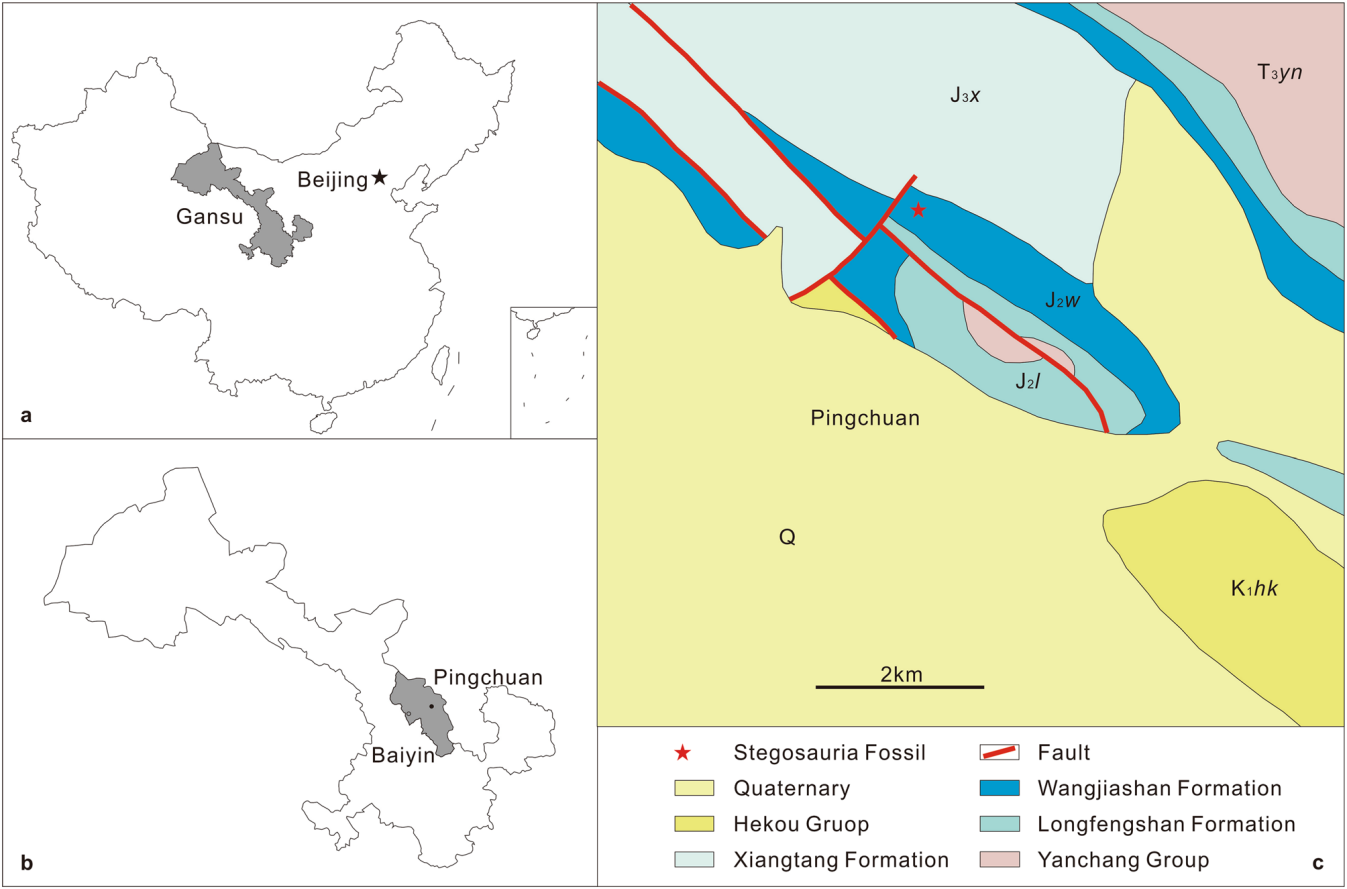
Locality and geological maps of the fossil locality. (**a**) map of China showing Gansu Province. (**b**) map of Gansu showing Baiyin City and Pingchuan District. (**c**) Geological map of Pingchuan District and the fossil locality.

The study area is located in the northwestern part of the Baojishan Basin, belonging to the Pinchuan District of Baiyin City, Gansu Province (Fig. 1a,b). The Baojishan Basin is at the eastern end of the Qilian Mountains. It is a fault-bounded subsiding basin, being a small part of the Hexi Corridor Basin, which developed in the Late Triassic and Jurassic. The Middle Jurassic strata of the area is composed of two lithostratigraphic units, the Longfengshan Formation and the Wangjiashan Formation. Underlying these Middle Jurassic strata is a disconformity with Upper Triassic rocks, while Upper Jurassic sediments conformably overlie them^26,27^. The Straw-yellow Sandstone Member of the Wangjiashan Formation was deposited mainly in a lake deltaic setting, and the succession is characterized by its straw-yellow colour. It changes gradually upward from coarse sandstones at the base to fine sands with interbedded red mudstones in the upper part. The dinosaur fossils were collected from the sandstone of the upper part of the Straw-yellow Sandstone Member of the Wangjiashan Formation (Fig. 1c). Based on its megafossil plants and palynoflora, as well as the lacustrine invertebrate assemblage of the overlying Oil Shale Member, including conchostracans, charophytes, and bivalves, the Straw-yellow Sandstone Member of the Wangjiashan Formation is considered to be late Bathonian in age^26–28^.

### Systematic palaeontology

Dinosauria Owen^29^

Ornithischia Seeley^30^

Thyreophora Nopcsa^31^

Stegosauria Marsh^32^

*Baiyinosaurus baojiensis* gen. et sp. nov.

#### Holotype

IVPG-D021. A partial skeleton comprising a partial cranium, one cervical vertebra, seven dorsal vertebrae and one caudal vertebra. The measurements of the vertebrae can be found in Table 1.

**Table 1:** Measurements (in mm) of vertebrae.

Element	Centrum length	Anterior facet width	Anterior facet height	Posterior facet width	Posterior facet height	Neural canal height	Neural arch height
Atlas (Fig. 4)	23	49	8	50	27	–	–
Second dorsal vertebra (Fig. 5e–j)	62	78	58	82	73	34	46
Third dorsal vertebra (Fig. 6a–f)	78	69	54	70	68	35	52
Fourth dorsal vertebra (Fig. 6a–f)	69	75	64	81	55	43	–
Fifth dorsal vertebra (Fig. 6g–l)	78	62	54	71	60	31	56
Sixth dorsal vertebra (Fig. 6m–r)	84	54	41	50	57	37	57
Seventh dorsal vertebra (Fig. 6s–x)	81	65	55	68	64	41	65
Caudal vertebra (Fig. 7a–f)	46	71	58	76	62	23	-

#### Etymology

Generic name is a combination of Baiyin (the city in which the type locality is located) and saurus (Greek, reptile). The specific name is derived from Baoji (the basin name of the type locality).

#### Locality and Horizon

Pingchuan District, Baiyin City, Gansu Province, China. GPS coordinates: N36° 44′ 59.1″, E104° 49′ 53.2″. The materials are from the upper part of the Straw-yellow Sandstone Member of the Wangjiashan Formation, whose age is considered to be Middle Jurassic, late Bathonian.

#### Diagnosis

*Baiyinosaurus* differs from all other stegosaurs by possession of the following autapomorphy: the frontal is wider than long (score 1 for character 32), and not only contributes to the medial margin of the supratemporal fenestra but also makes up a very great contribution to the anterior margin of the supratemporal fenestra. *Baiyinosaurus* also possesses the following character combination on dorsal vertebrae: (1) the neural arches are not greatly elongated dorsally: the ratio of neural arch height to neural canal height is 1.59 (character 6, a continuous character); (2) the parapophyses are well developed and project somewhat laterally on stalks in anterior view (score 0 for character 64); (3) the neural spines are anteroposteriorly broad and axially expanded in lateral view: the ratio of neural spine length (measured at the base) to centrum length is 0.75 (character 9, a continuous character).

### Description and comparisons

#### Skull

**Figure 2:**
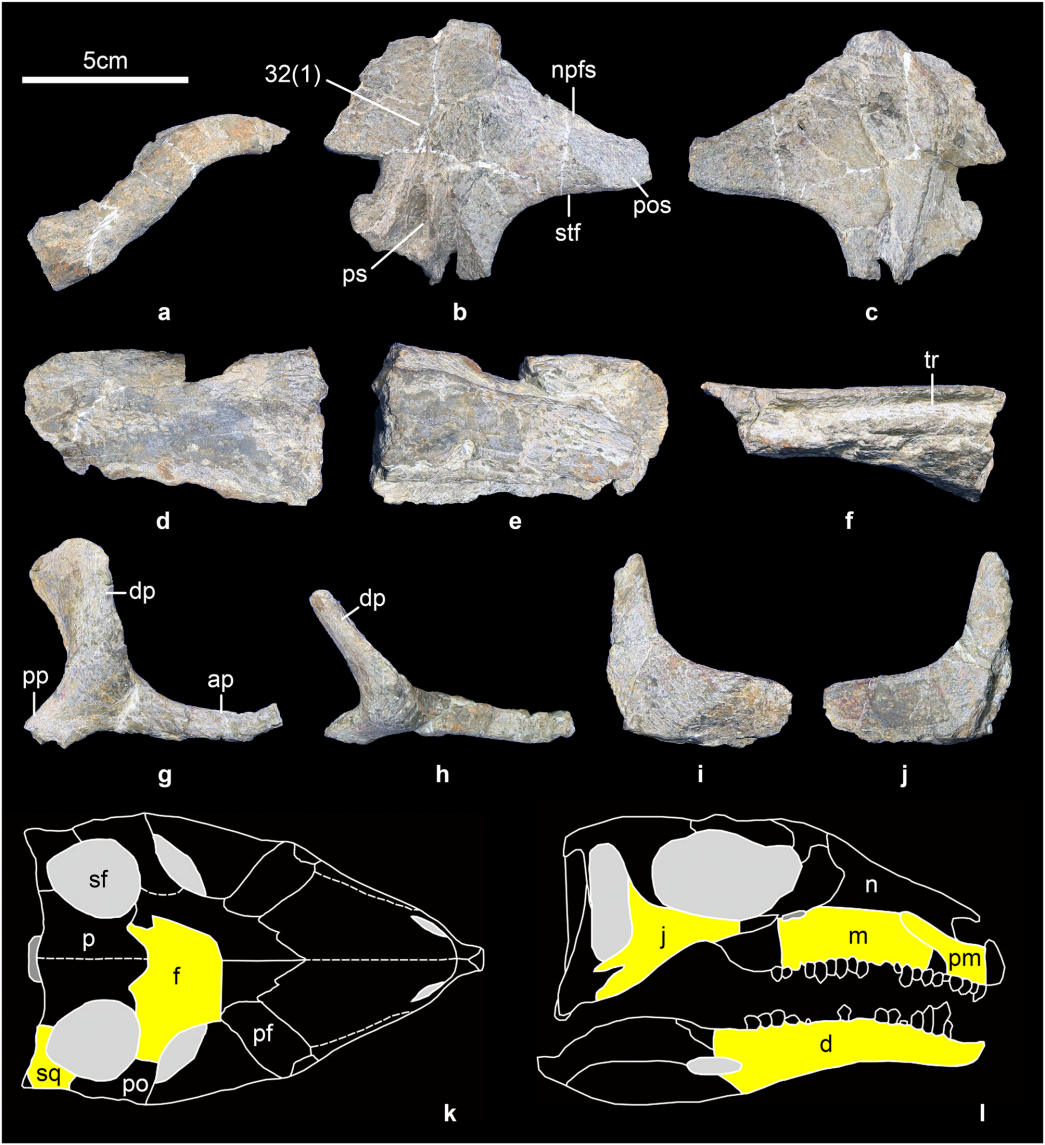
Skull of *Baiyinosaurus baojiensis*. (**a**) left premaxilla (IVPG-D021-01), (**b, c**) frontal (IVPG-D021-02), (**d**–**f**) left maxilla (IVPG-D021-03), (**g, h**) right jugal (IVPG-D021-04), (**i, j**) right squamosal (IVPG-D021-05). (**a**, **d**, **g**) lateral view, (**b**, **h**, **i**) dorsal view, (**c**, **f**, **j**) ventral view, (**e**), medial view. Reconstruction of the skull of *Baiyinosaurus* in dorsal (**k**) and lateral (**l**), modified from *Emausaurus*^33^. The yellow areas represent preserved bones. *Ap* anterior process, *d* dentary, *dp* dorsal process, *f* frontal, *j* jugal, *m* maxilla, *n* nasal, *npfs* nasal and prefrontal suture, *p* parietal, *pf* prefrontal, *pm* premaxilla, *po* postorbital, *pos* postorbital suture, *pp* posterior process, *ps* parietal suture, *sf* supratemporal fossae, *sq* squamosal, *stf* margin of supratemporal fenestra, *tr* teeth row.

Some parts of the skull are preserved incompletely, including the left premaxilla (Fig. 2a), frontal (Fig. 2b,c), left maxilla (Fig. 2d–f), right jugal (Fig. 2g,h), and right squamosal (Fig. 2i,j). The skull of *Baiyinosaurus* is reconstructed in Fig. 2k,l, which is modified from *Emausaurus*^33^.

Premaxilla: only the posterior part of the left premaxilla is preserved (Fig. 2a). It is an oblique, slightly sinuous, gracile posterolateral process that wedges itself between the maxilla and nasal, similar to *Stegosaurus*^34^, *Scelidosaurus*^35^ and *Emausaurus*^33^. The dorsal margin of the posterolateral process has an obvious transversely expanded contact surface for the nasal.

Maxilla: the anterior and middle parts of the left maxilla are nearly completely preserved, but the anteroventral and posterior parts are missing. The lateral surface of the maxilla is flat (Fig. 2d). The complete tooth row is inset medially from the lateral surface (Fig. 2f), similar to other stegosaurs and most other ornithischians^36,37^. The dorsolateral surface of the maxilla is laterally compressed, forming a thin lamina that forms the lateral wall of the nasal cavity (Fig. 2e).

Frontal: although the frontal is incomplete (Fig. 2b,c), its overall form is shorter anteroposteriorly than most stegosaurs, but is similar to *Tuojiangosaurus*^38^ and the basally branching thyreophorans *Scelidosaurus*^35^ and *Emausaurus*^33^. It is flat in dorsal view. Posteriorly, the sutural boundaries overlapped by the parietal are clear (Fig. 2b). The right part of the frontal is almost complete and the structure overlapped by the postorbital is visible (Fig. 2b). The end of this structure is narrower anteroposteriorly than *Huayangosaurus*^39^ and *Stegosaurus*^34^, but more similar to *Emausaurus*^33^. Right laterally, the anterior margin has a structure overlapped by the nasal and the prefrontal, and the posterior margin is smooth forming the supratemporal fossae (Fig. 2b). The frontal not only contributes to the medial margin of the supratemporal fenestra but also makes up a much greater contribution to the anterior margin of the supratemporal fenestra than in other stegosaurs, such as *Huayangosaurus*^39^, *Stegosaurus*^34^ and *Kentrosaurus*^40^.

Jugal: a possible jugal is present. The jugal (Fig. 2g,h) is triradiate and contacts postorbital dorsally, maxilla anteriorly and quadratojugal posteroventrally. The end of the anterior process slightly curved dorsally because of fracture. The dorsal process is very broad and extends medially.

Squamosal: a fragment that may represent the dorsal part of the right squamosal is preserved (Fig. 2i,j). It is flat on the dorsal surface and concave on the ventral surface. In dorsal view, the left part has a suture overlapped by the parietal and the right part has a structure overlapped by the postorbital.

#### Dentary

**Figure 3:**
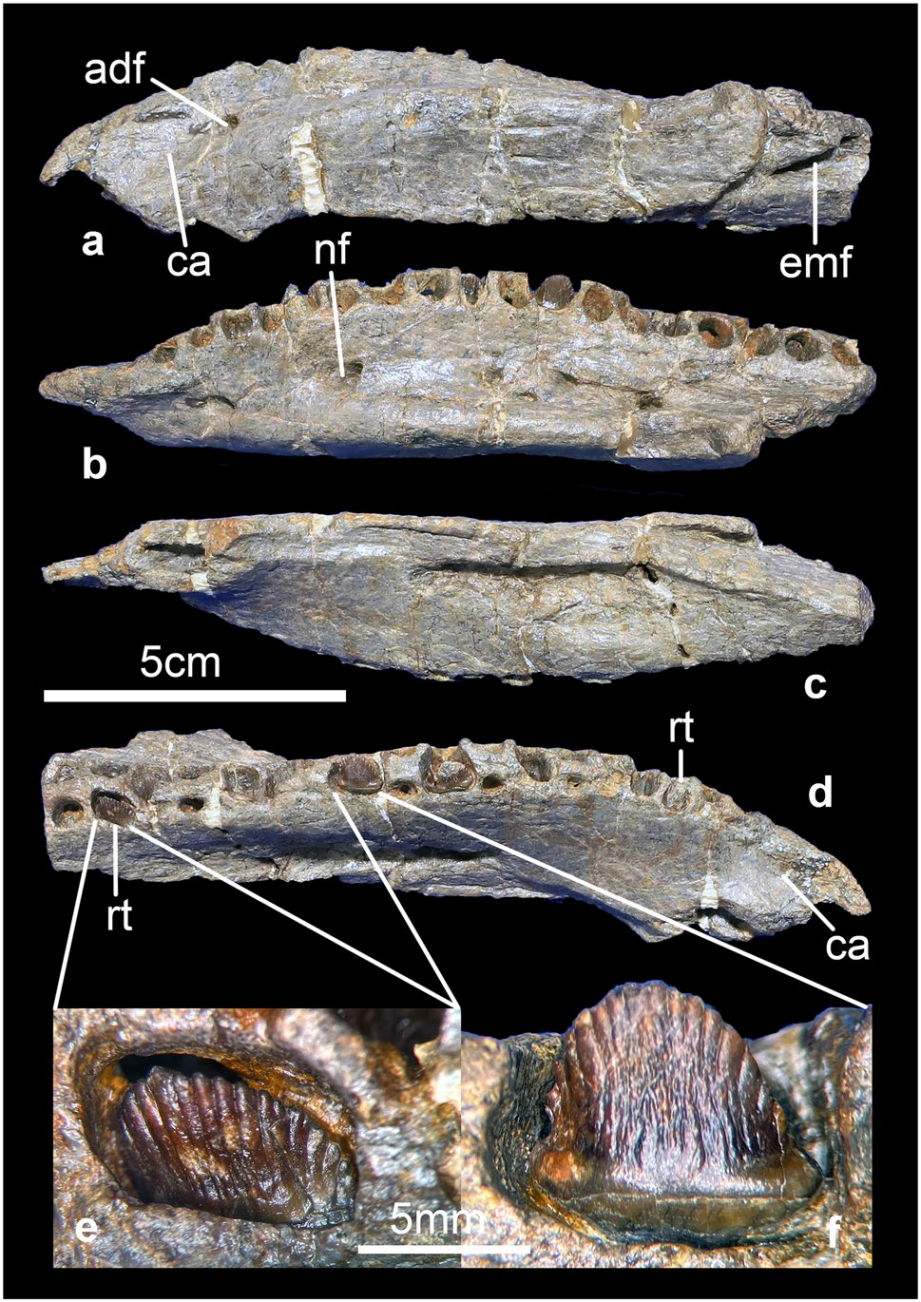
Dentary of *Baiyinosaurus baojiensis* (IVPG-D021-06). (**a–d**) left dentary, (**e–f**) teeth. (**a**) lateral view, (**b**) dorsal view, (**c**) ventral view, (**d**) medial view, (**e**, **f**) lingual view. *Adf* anterior dentary foramen, *ca* concave area, *emf* external mandibular fenestra, *nf* nutrient foramina, *rt* replacing teeth.

The left dentary is almost complete and curves medioventrally (Fig. 3a–d). Anteriorly, there are concave facets for the predentary on both the medial and lateral surfaces (Fig. 3a,d). The first dentary tooth arises immediately posterior to these facets for the predentary, with no diastema, similar to the condition in *Huayangosaurus*^39^, *Gigantspinosaurus* (ZDM 0019) and *Kentrosaurus* (MB.R.3806.1), but in contrast to *Stegosaurus*^41^ and *Jiangjunosaurus*^42^, where there is a diastema of several tooth-widths between the predentary and the first dentary tooth. A large vascular foramen, the anterior dentary foramen, opens laterally on the dentary and posterior to the predentary area (Fig. 3a). In lateral, the tooth row is visible and sinuous, as in *Huayangosaurus*^39^,* Gigantspinosaurus* (ZDM 0019) and *Jiangjunosaurus*^42^ but different to *Stegosaurus*^34^, where a lateral lamina in lateral view obscures the tooth row. An external mandibular fenestra is present at the posterior end of the dentary (Fig. 3a). In dorsal view, the tooth row is strongly offset medially and the tooth alveoli face dorsomedially, similar to *Stegosaurus*^34^ and *Gigantspinosaurus*^43^. There is a row of nutrient foramina lateral to the tooth row in both dorsal and ventral views. The dentary contains eighteen alveoli, in some of which there are teeth in various stages of eruption. In medial view, replacement teeth within resorption pits, lingual to the empty alveoli, are present.

#### Teeth

The two complete teeth have seven denticles on either side of two apical denticles (Fig. 3e,f). *Tuojiangosaurus* also has seven denticles on each side of the tooth crown^44^. In *Stegosaurus*, the number of denticles is variable between teeth, and varies between five and nine on either side of two to four apical denticles^34^ (NHMUK PV R 36730), while in *Paranthodon* there are four to five denticles on either side of a central one^45^ (NHMUK PV OR 47338; NHMUK PV R 4992). Striations on the tooth crown are not confluent with marginal denticles, as in most stegosaurs except *Paranthodon*^46^. The cingulum is present similar to other stegosaurs except *Huayangosaurus*, where a swelling at the base of the crown is present, but this is not developed into a distinct cingulum^39^. Compared to the replacement tooth (Fig. 3e), the functional tooth is slightly worn (Fig. 3f), and its wear facet is inclined anteroventrally or posteroventrally. The microwear of the tooth is similar to *Kentrosaurus*^40^, but some stegosaurs have a higher degree of tooth wear, such as in *Huayangosaurus*^39^ and *Gigantspinosaurus*^43^, and especially the stegosaurian teeth discovered in Montana, USA^47^ and Yakutia, Eastern Russia^48^. The higher degree of tooth wear may indicate that the tooth itself is more mature and does not represent an individual’s maturational status^47^.

#### Cervical vertebra

**Figure 4:**
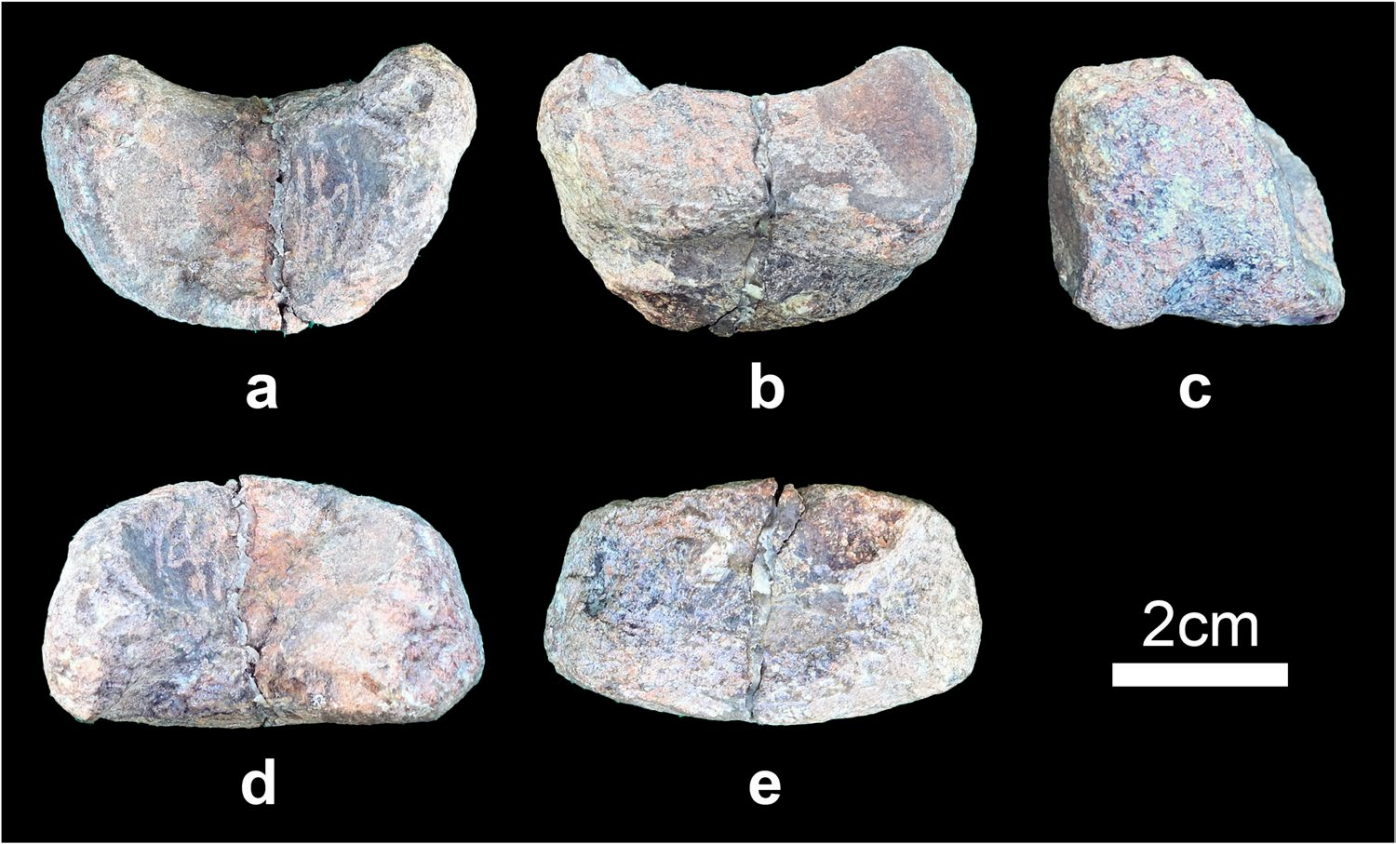
Atlas of *Baiyinosaurus baojiensis* (IVPG-D021-07). (**a**) anterior, (**b**) posterior, (**c**) right lateral, (**d**) dorsal view, (**e**) ventral view.

The only cervical vertebra preserved is the atlas (Fig. 4a–e). The atlas only preserves the intercentrum. It is saddle-shaped in anterior view (Fig. 4a) and its posterior margin is elevated dorsally in posterior view (Fig. 4b). In *Stegosaurus*, there are two ridges on the ventral surface of the intercentrum^49^ which are not present in *Baiyinosaurus*.

#### Dorsal vertebrae

**Figure 5:**
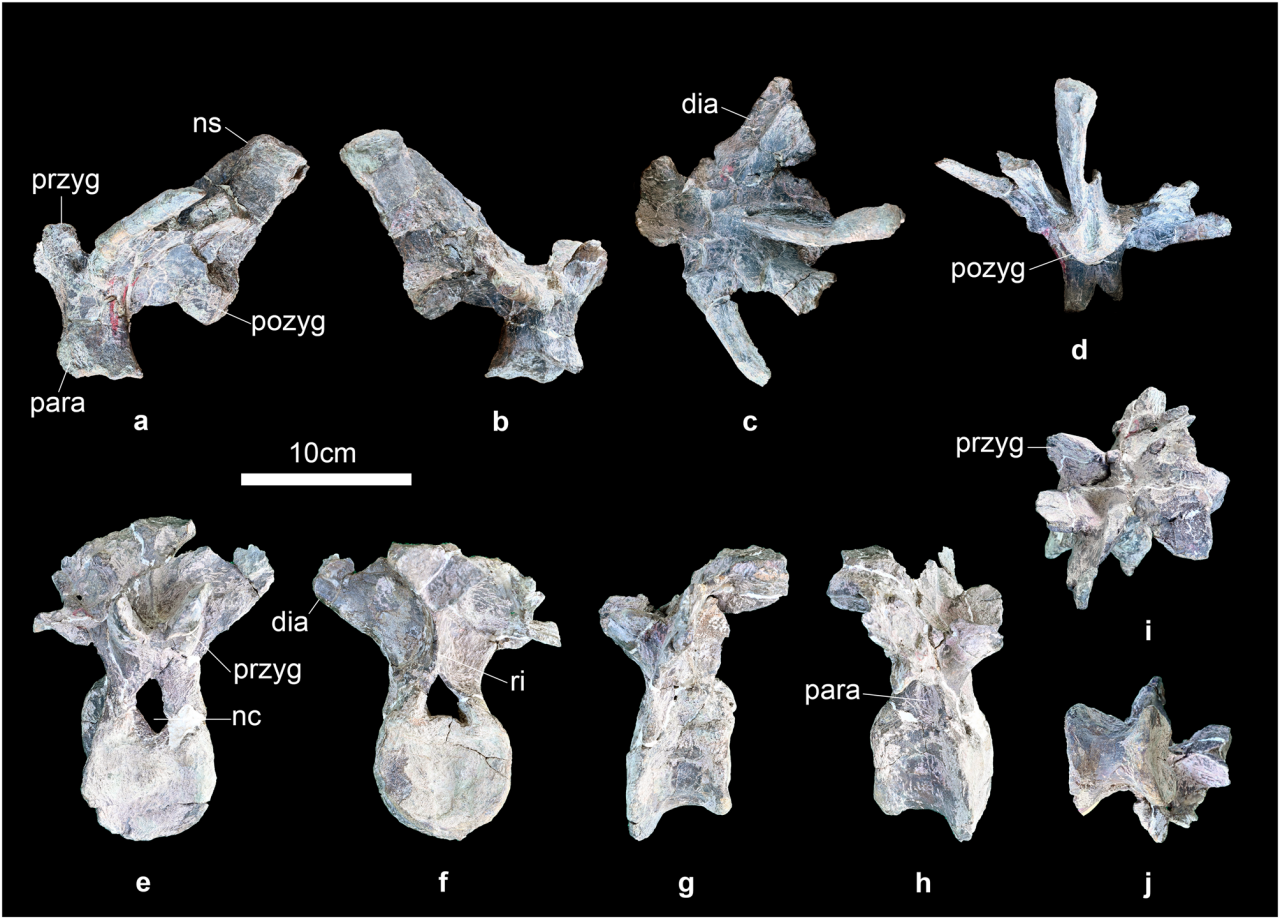
(**a**–**j**) anterior dorsal vertebrae of *Baiyinosaurus baojiensis* (IVPG-D021-08 and IVPG-D021-09). (**a**, **g**) left lateral, (**b**, **h**) right lateral, (**c**, **i**) dorsal view, (**d**, **f**) posterior, (**e**) anterior, (**j**) ventral view. *Dia* diapophysis, *nc* neural canal, *ns* neural spine, *para* parapophysis, *pozyg* postzygapophysis, *przyg* prezygapophysis, *ri* ridge.

**Figure 6:**
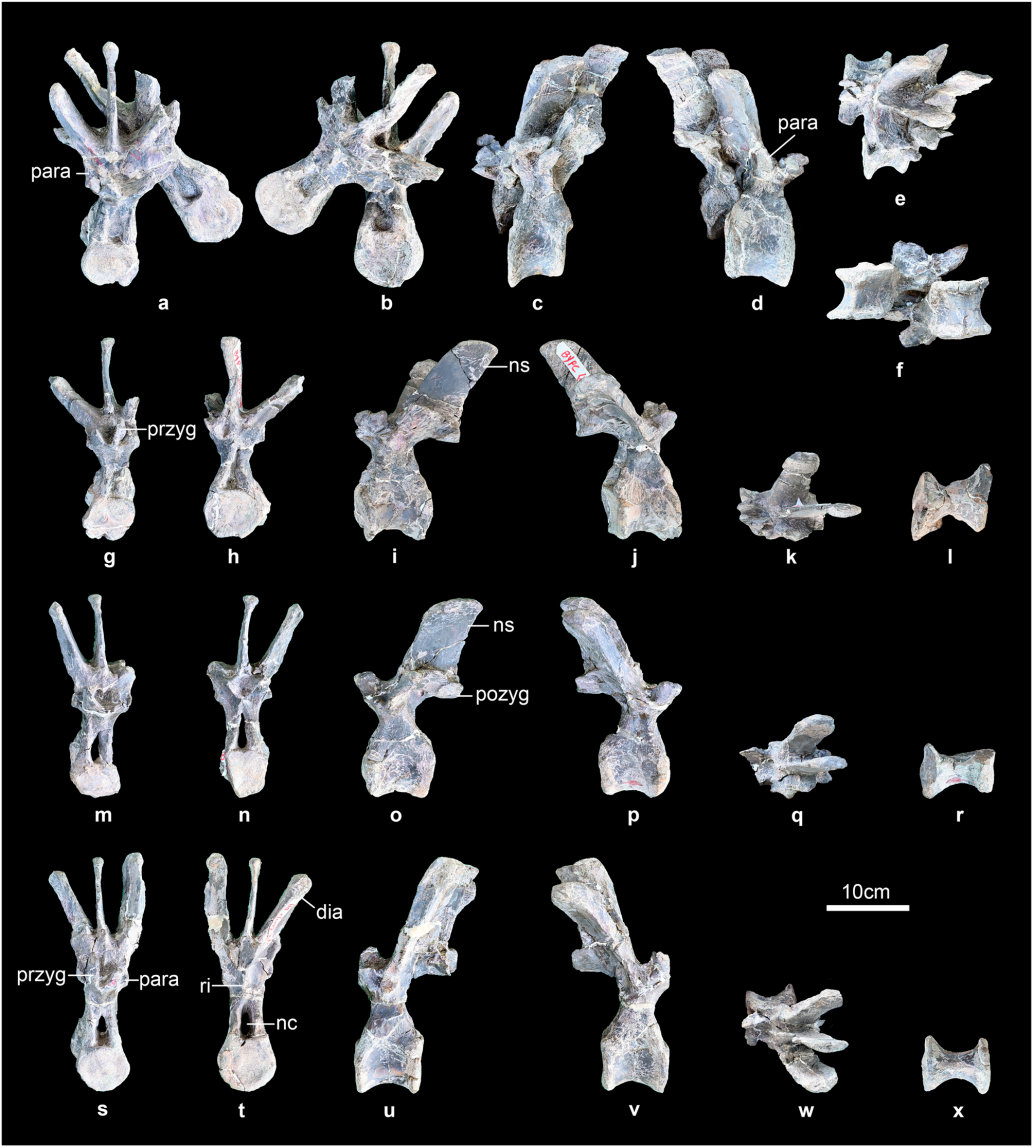
(**a**–**x**) middle or posterior dorsal vertebrae of *Baiyinosaurus baojiensis* (IVPG-D021-10 to IVPG-D021-14). (**a**, **g**, **m**, **s**) anterior, (**b**, **h**, **n**, **t**) posterior, (**c**, **i**, **o**, **u**) left lateral, (**d**, **j**, **p**, **v**) right lateral, (**e**, **k**, **q**, **w**) dorsal view, (**f**, **l**, **r**, **x**) ventral view. *Dia* diapophysis, *nc* neural canal, *ns* neural spine, *para* parapophysis, *pozyg* postzygapophysis, *przyg* prezygapophysis, *ri* ridge.

Seven dorsal vertebrae are preserved including two anterior dorsal vertebrae (Fig. 5a–j) and five middle or posterior dorsal vertebrae (Fig. 6a–x). Only one dorsal vertebra is preserved completely (Fig. 6s–x), and two of the dorsal vertebrae are associated (Fig. 6a–f). The centra of all these dorsal vertebrae are almost amphiplatyan, similar to most stegosaurs. Both the anterior and posterior articular surfaces of the centra are round in outline. The centrum is wider transversely than long anteroposteriorly in the anterior dorsal vertebra (Fig. 5e–h), but are longer than wide in middle and posterior dorsal vertebrae (Fig. 6a–x). The ventral surfaces of the centra are smooth and the ventral margin of the centra is gently concave upwards in lateral view. The neural arch is not greatly expanded dorsally, similar to early-diverging stegosaurs such as *Huayangosaurus*^50^ and *Gigantspinosaurus*^43^, but different to most other stegosaurs such as *Stegosaurus*^49^, *Kentrosaurus*^51^ and *Tuojiangosaurus*^44^. The neural canal has a sub-ovate outline in anterior and posterior views. The parapophyses of the dorsal vertebra (IVPG-D021-08) are situated at the base of the neural arch and the parapophyses of the dorsal vertebra (IVPG-D021-09) have migrated entirely onto the lateral surface of the neural arch, which is similar to the first and second dorsal vertebrae of *Stegosaurus*^49^. However, the parapophyses of the third dorsal vertebra of *Stegosaurus*^49^ have migrated onto the upper part of the neural arch, indicating the two anterior dorsal vertebrae of *Baiyinosaurus* may be the first and second dorsal vertebrae. The parapophyses of five middle or posterior dorsal vertebrae are situated at the base of the diapophyses. In the anterior dorsal vertebrae, the parapophysis has a concave articular surface (Fig. 5a,h). However, the parapophysis is elevated on a short stalk in middle or posterior dorsal vertebrae (Fig. 6a,g,m,s), similar to the early-diverging stegosaur *Bashanosaurus*^6^. In the anterior, the prezygapophyses are joined ventrally and face dorsomedially. The articular surfaces of prezygapophyses are flat. In anterior view, the diapophyses extend dorsolaterally at a high angle to the horizontal, similar to most stegosaurs such as *Stegosaurus*^49^ and *Huayangosaurus*^50^. From the anterior to posterior dorsal vertebrae, the angle to the horizontal of the diapophyses increases. The diapophysis is compressed dorsoventrally. In posterior view, the postzygapophyses are triangular in outline and linked to the neural canal by a ridge, similar to *Stegosaurus*^49^. The neural spine projects posterodorsally in lateral view and is a transversely compressed plate in anterior view. The neural spine is significantly elongated anteroposteriorly, similar to *Gigantspinosaurus*^43^, but this is developed to a greater degree than in most stegosaurs such as *Huayangosaurus*^50^, *Stegosaurus*^49^ and *Bashanosaurus*^6^.

#### Caudal vertebra

**Figure 7:**
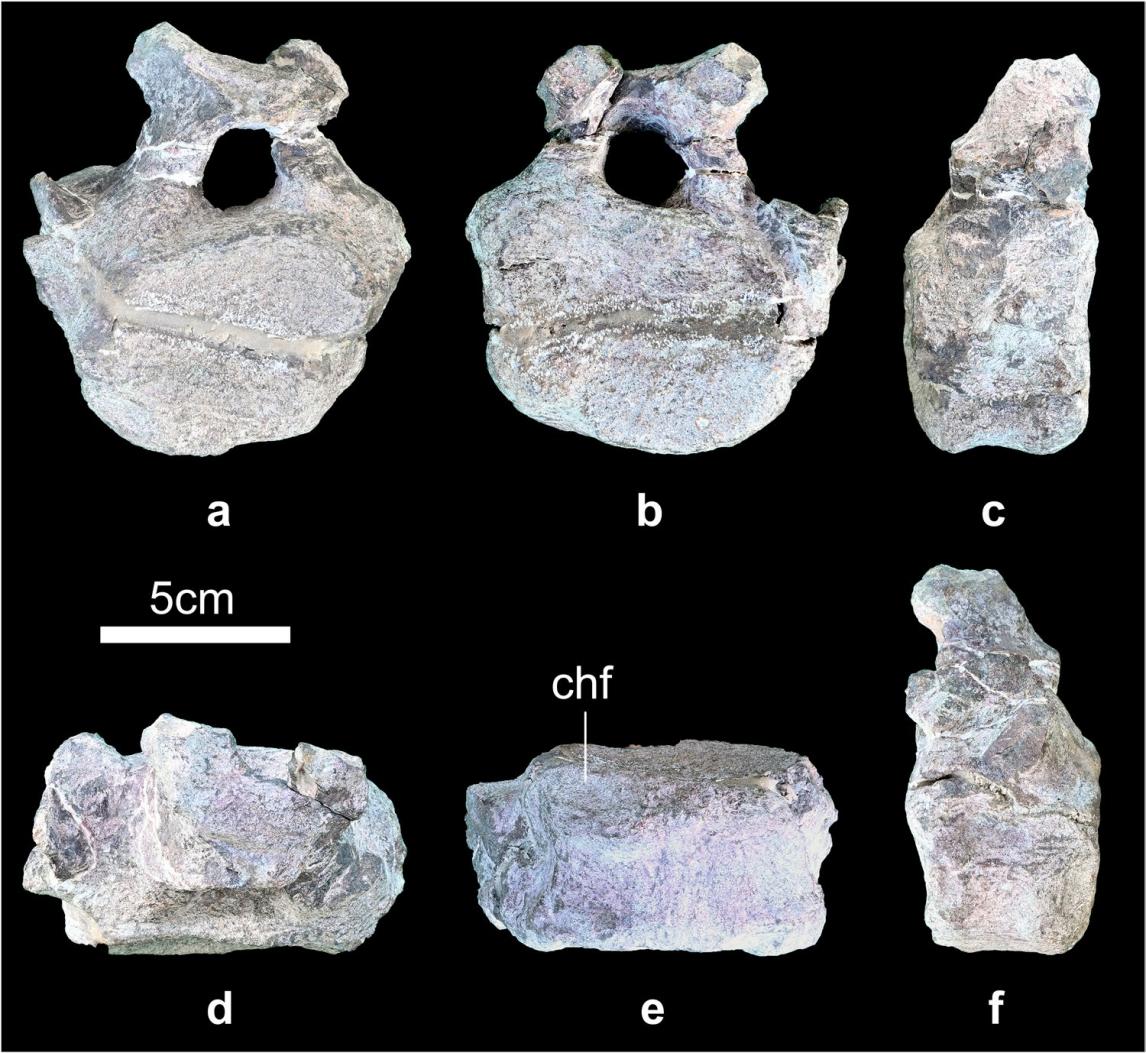
Anterior caudal vertebra of *Baiyinosaurus baojiensis* (IVPG-D021-15). (**a**) anterior, (**b**) posterior, (**c**) left lateral, (**d**) dorsal view, (**e**) ventral view, (**f**) right lateral. *chf* chevron facet**.**

One anterior caudal vertebra is preserved but it is incomplete (Fig. 7a–f). Only the bases of the transverse processes are preserved, and the prezygapophyses, postzygapophyses and neural spine are missing. The anterior and posterior articular surfaces of the centrum are flat and round in outline. The anterior part of the ventral surface of the centrum has a chevron facet (Fig. 7e). The neural canal is round in outline.

### Phylogenetic analysis

**Figure 8:**
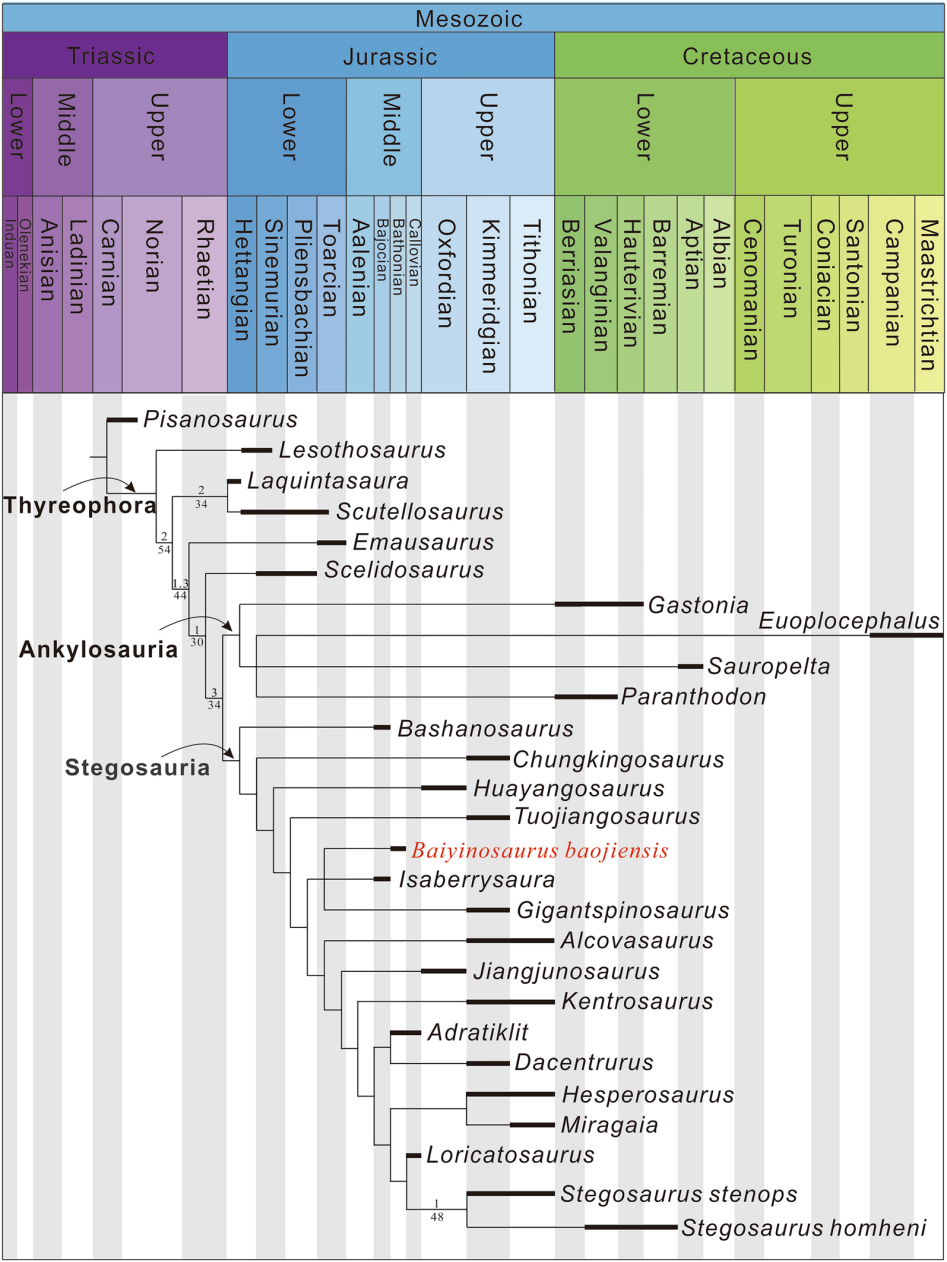
A single most parsimonious tree recovered by the phylogenetic analysis. Bremer supports no less than 1 and bootstrap support percentages are indicated above and below the line. Modified from Dai^6^.

A single most parsimonious tree was recovered (Fig. 8). The MPT was 276.06 steps in length and had a consistency index of 0.598 and a retention index of 0.659. *Baiyinosaurus* is recovered in a clade with *Isaberrysaura* and *Gigantspinosaurus*, albeit with low Bremer supports and bootstrap frequencies. Although *Paranthodon* is generally considered to be a stegosaur, it was found in Ankylosauria in this study. This result may be due to its fragmentary state and highly labile phylogenetic position^45^.

## Discussion

**Figure 9:**
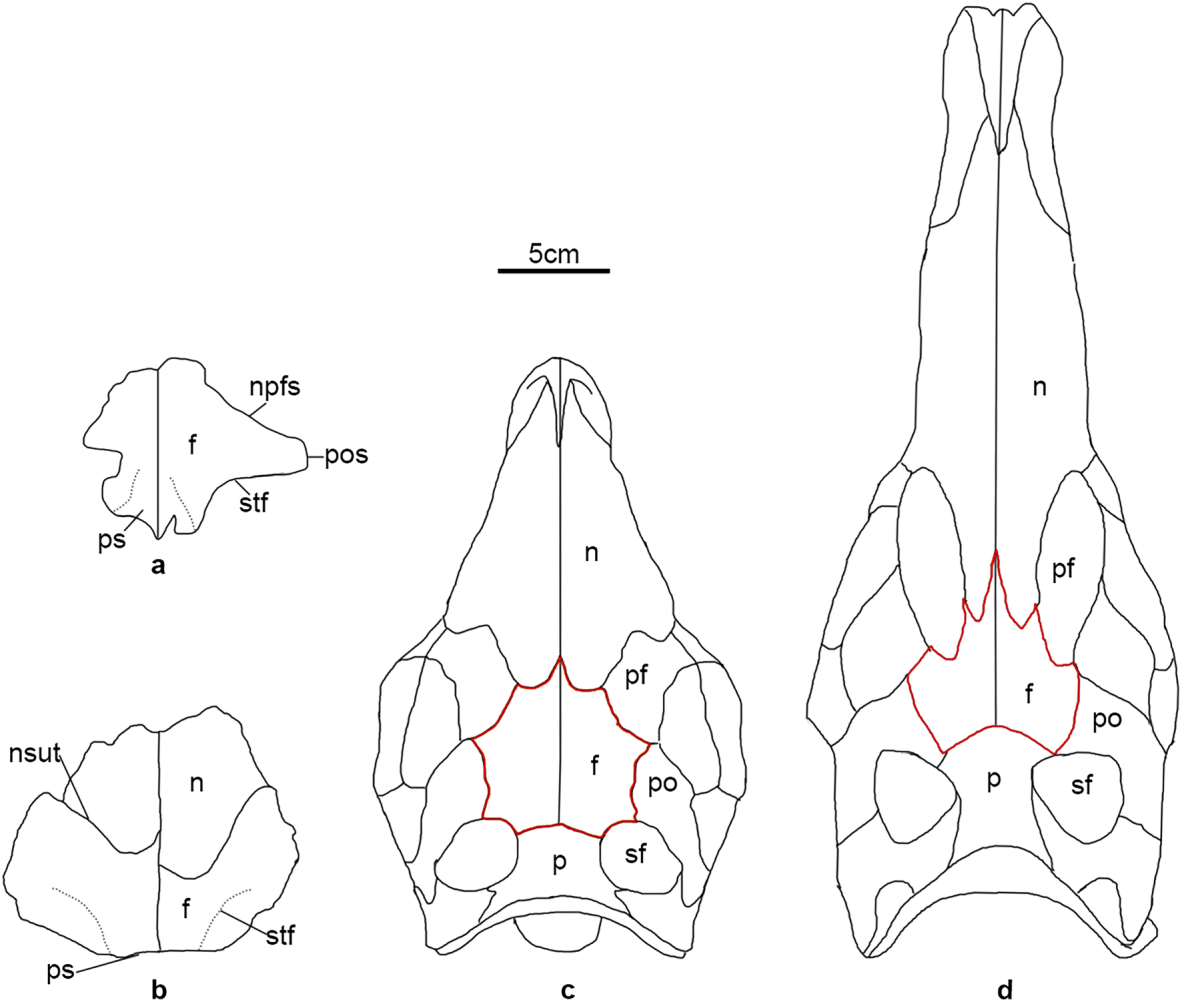
Outline of the frontal and other skull elements in dorsal view. (**a**) *Baiyinosaurus*, (**b**) *Tuojiangosaurus* (modified from Maidment and Wei^38^), (**c**) *Hauyangosaurus* (modified from Sereno and Dong^39^), (**d**) *Stegosaurus* (modified from Galton and Upchurch^1^). *F* frontal, *n* nasal, *npfs* nasal and prefrontal suture, *nsut* nasal suture; *p* parietal, *pf* prefrontal, *po* postorbital, *pos* postorbital suture, *ps* parietal suture, *sf* supratemporal fossae, *sq* squamosal, *stf* margin of supratemporal fenestra.

The neurocentral sutures on the dorsal vertebrae are invisible, indicating *Baiyinosaurus* probably is adult^52^. Unfortunately, due to a paucity of other bones, we are not able to adequately assess the ontogenetic stage of *Baiyinosaurus*. Although *Baiyinosaurus* is clearly a stegosaur based on the elongate neural arch pedicel in dorsal vertebrae and the diapophyses of dorsal vertebrae projecting at a high angle to the horizontal, it has a significant difference from other stegosaurs in the morphology of the skull. The frontal of the *Baiyinosaurus* is wider than long, different to most stegosaurs except *Tuojiangosaurus*^38^ (Fig. 9a–d). However, the frontal of the *Baiyinosaurus* not only contributes to the medial margin of the supratemporal fenestra but also makes up a much greater contribution to the anterior margin of the supratemporal fenestra than in other taxa, such as *Tuojiangosaurus*^38^, *Huayangosaurus*^39^ and *Stegosaurus*^34^. Besides, *Baiyinosaurus* has some plesiomorphic characteristics in the vertebrae shared with early-diverging thyreophorans and early-diverging stegosaurs: its neural arch is not greatly expanded dorsally, similar to basally-branching thyreophorans and early-diverging stegosaurs such as *Bashanosaurus*^6^, *Gigantspinosaurus*^43^ and *Hauyangosaurus*^53^. Its parapophysis is well developed and elevated on a short stalk, similar to the early-diverging stegosaur *Bashanosaurus*^6^ and the basally branching thyreophoran *Scelidosaurus*^54^. It has broad, axially expanded neural spine in lateral view, similar to the condition in the early-diverging stegosaur *Gigantspinosaurus*^43^ and the basally branching thyreophorans *Scelidosaurus*^54^, *Laquintasaura*^55^ and *Lesothosaurus*^56^. These character combinations of dorsal vertebrae of *Baiyinosaurus* are also different to other stegosaurs.

The diapophyses of the dorsal vertebrae of stegosaurs projecting dorsolaterally at a high angle to the horizontal is one of the most obvious differences compared to basally branching thyreophorans. *Baiyinosaurus* and other early-diverging stegosaurs such as *Bashanosaurus*^6^ and *Gigantspinosaurus*^43^ show that features generally considered to be characteristic of stegosaurs, such as the elongated neural arch and parapophyses located at the base of the diapophyses as flattened, oval facets were acquired gradually during stegosaur evolution. First, there was a gradual increase in the angle of projection of the diapophyses of the dorsal vertebrae: they project horizontally in basally branching thyreophorans such as *Scutellosaurus*^57^ and *Laquintasaura*^55^, slightly dorsolaterally in *Scelidosaurus*^54^ and project dorsolaterally at a high angle in stegosaurs, even in the early-diverging stegosaurs *Baiyinosaurus* and *Bashanosaurus*^6^. The neural spine changed from anteroposteriorly broad to being narrower anteroposteriorly during thyreophoran evolution: the neural spine is axially expanded in basally-branching thyreophorans such as *Scelidosaurus*^54^, *Laquintasaura*^55^ and *Lesothosaurus*^56^, and early-diverging stegosaurs such as *Baiyinosaurus*, but is narrower in the early-diverging stegosaur *Bashanosaurus*^6^ and other stegosaurs. Finally, the parapophysis was elevated on a short stalk in the basally branching thyreophoran *Scelidosaurus*^54^, and early-diverging stegosaurs *Baiyinosaurus* and *Bashanosaurus*^6^, but is not elevated in later-diverging stegosaurs, and is instead present as a facet on the lateral surface of the neural arch ventral to the diapophysis.

The basally-branching thyreophorans, the ancestors of the stegosaurs, are all from the Early Jurassic, including *Scelidosaurus*^54^ from England, *Emausaurus*^33^ from Germany, *Lesothosaurus*^56,58,59^ from South Africa, *Scutellosaurus*^57,60,61^ from the United States, *Laquintasaura*^55^ from Venezuela and the poorly known *Tatisaurus*^62,63^ and *Bienosaurus*^64,65^ from China. Although the earliest tracks of possible stegosaurs are from the Early Jurassic^1^, the earliest bones of stegosaurs are found in the Middle Jurassic^4^. *Baiyinosaurus* has some character states that indicate it was transitional in morphology between the basally branching thyreophorans and the stegosaurs, and is also from the Middle Jurassic, indicating a Middle Jurassic (or much earlier) origin for the split between Stegosauria and its sister taxon Ankylosauria is likely.

## Conclusions

*Baiyinosaurus* is a new taxon and represents one of the earliest records of Stegosauria. Systematic analysis shows that *Baiyinosaurus* is an early-diverging stegosaur closed to *Isaberrysaura*, *Alcovasaurus*, *Jiangjunosaurus* and *Gigantspinosaurus*. *Baiyinosaurus* has some plesiomorphic characteristics and is transitional in morphology between early thyreophorans and early-diverging stegosaurs. The increasing diversity of Middle Jurassic stegosaurs that also occupy early-diverging positions indicates that it is likely that the stegosaurs split from their sister taxon, the ankylosaurs, early in the Middle Jurassic (or much earlier).

## Methods

Phylogenetic analysis. To assess the phylogenetic position of *Baiyinosaurus*, it is added to the character-taxon matrix of Dai et al.^6^. The matrix (updated from Maidment et al.^4^) is based on Raven and Maidment^66^ which originally comprised 23 taxa scored for 115 characters. We added two new characters to the dataset: 9. Dorsal vertebrae: neural spines length (measured at the base) to centrum length ratio coded continuously. 64. Dorsal vertebrae: parapophyses are well developed that held on stalks at the base of the diapophyses; (0); poorly developed (1). The data matrix consists of 27 taxa scored for 117 morphological characters. The character list and data matrix can be found in supplementary A and B. The matrix was analyzed in TNT v1.5^67^. *Pisanosaurus*^68^ was set as the outgroup. All continuous characters (1–25) and characters 108 and 109 were ordered. A New Technology search was performed using sectorial, ratchet, drift and tree fusing options and 10 random addition sequences. The most parsimonious trees (MPTs) recovered from the New Technology search were used as the starting point for a round of tree bisection-reconnection (TBR) using the Traditional Search option with one random addition seed and 1000 replicates. Support for the relationships obtained was evaluated using Bremer support and bootstrap analysis (1000 replicates, traditional search). Nomenclatural acts: The nomenclatural acts it contains have been registered in ZooBank, the online registration system for the ICZN. The ZooBank LSIDs (Life Science Identifiers) can be resolved and the associated information viewed through any standard web browser by appending the LSID to the prefix ‘http://zoobank.org/’. The LSID for this publication is: urn:lsid:zoobank.org:pub:5A2665F4-F3D0-4519-808C-E4CEC8E07E00.

### Supplementary Information

Supplementary Information 1.

Supplementary Information 2.

## Data Availability

All data generated or analysed during this study are included in this published article and its supplementary information fles.

## Abbreviations

IVPG: Institute of Vertebrate Paleontology, Gansu Agricultural University
MB: Museum für Naturkunde, Berlin, Germany
NHMUK: Natural History Museum, London, UK
ZDM: Zigong Dinosaur Museum, Zigong, Sichuan Province, People’s Republic of China

## References

[1] Galton, P. M. & Upchurch, P. Stegosauria. In *The Dinosauria (2nd edition)* (eds. Weishampel D. B. et al.) 343–362 (University of California Press, 2004).

[2] Nopcsa F (1911). Notes on British dinosaurs IV: Stegosaurus priscus sp. nov. Geol. Mag..

[3] Salgado L, Canudo JI, Garrido AC, Moreno-Azanza M, Martínez LCA, Coria RA (2017). A new primitive Neornithischian dinosaur from the Jurassic of Patagonia with gut contents. Sci. Rep.-UK.

[4] Maidment SCR, Raven TJ, Ouarhache D, Barrett PM (2020). North Africa's first stegosaur: Implications for Gondwanan thyreophoran dinosaur diversity. Gondwana Res..

[5] Dong ZM, Tang ZL, Zhou SW (1982). Note on the new Mid-Jurassic stegosaur from Sichuan Basin, China. Vertebr. Palasiat..

[6] Dai H, Li N, Maidment SCR, Wei GB, Zhou YX, Hu XF (2022). New Stegosaurs from the Middle Jurassic Lower member of the Shaximiao formation of Chongqing. China. J. Vertebr. Paleontol..

[7] Bohlin B (1953). Fossil reptiles from Mongolia and Kansu. Sino-Swedish Exped. Publ..

[8] You HL, Tang F, Luo ZX (2003). A new basal titanosaur (Dinosauria: Sauropoda) from the Early Cretaceous of China. Acta Geol. Sin..

[9] You HL, Li DQ, Zhou LQ, Ji Q (2006). *Huanghetitan liujiaxiaensis*, a new sauropod dinosaur from the Lower Cretaceous Hekou Group of Lanzhou Basin, Gansu Province, China. Geol. Rev..

[10] You HL, Li DQ, Zhou L, Ji Q (2008). *Daxiatitan Binglingi*: A giant Sauropod dinosaur from the Early Cretaceous of China. Gansu Geol..

[11] You HL, Li DQ (2009). The first well-preserved Early Cretaceous brachiosaurid dinosaur in Asia. Proc. R. Soc. B Biol. Sci..

[12] Li LG, Li DQ, You HL, Dodson P, Butler RJ (2014). A new titanosaurian sauropod from the Hekou Group (Lower Cretaceous) of the Lanzhou - Minhe Basin, Gansu Province, China. Plos One.

[13] Li DQ, Peng C, You HL, Matthew CL, Jerald DH, Kenneth JL (2007). A large Therizinosauroid (Dinosauria:Theropoda) from the Early Cretaceous of Northwestern China. Acta Geol. Sin..

[14] Li DQ, Norell MA, Gao KQ, Smith ND, Makovicky PJ (2010). A longirostrine tyrannosauroid from the Early Cretaceous of China. Proc. R. Soc. B Biol. Sci..

[15] Makovicky PJ, Li DQ, Gao KQ, Lewin M, Erickson GM, Norell MA (2010). A giant ornithomimosaur from the Early Cretaceous of China. Proc. R. Soc. B Biol. Sci..

[16] Lü, J. C. A New Iguanodontidae (Probactrosaurus mazongshanensis sp. nov.) from Mazongshan Area, Gansu Province, China. In *Sino-Japanese Silk Road Dinosaur Expedition* (ed. Dong Z. M.) 27–47 (China Ocean Press, 1997).

[17] You HL, Peter D (2003). Redescription of neoceratopsian dinosaur Archaeoceratops and early evolution of Neoceratopsia. Acta Palaeontol. Pol..

[18] You HL, Luo ZX, Shubin NH, Witmer LM, Tang ZL, Tang F (2003). The earliest-known duck-billed dinosaur from deposits of late Early Cretaceous age in northwest China and hadrosaur evolution. Cretac. Res..

[19] You HL, Li DQ, Ji Q, Matthew CL, Peter D (2005). On a new genus of basal neoceratopsian dinosaur from the Early Cretaceous of Gansu Province, China. Acta Geol. Sin..

[20] You HL, Ji Q, Li DQ (2005). *Lanzhousaurus magnidens* gen. et sp. Nov. from Gansu Province, China; the largest-toothed herbivorous dinosaur in the world. Geol. Bull. China.

[21] You HL, Li DQ (2009). A new basal hadrosauriform dinosaur (Ornithischia: Iguanodontia) from the Early Cretaceous of northwestern China. Can. J. Earth Sci..

[22] You, H. L., Tanoue K & Dodson, P. A new species of Archaeoceratops (Dinoasuria: Neoceratopsia) from the Early Cretaceous of the Mazongshan Area, northwestern China. In *New perspectives on Horned Dinosaurs* (eds. Ryan M. J. et al.) 59–67 (Indiana University Press, 2010).

[23] You HL, Li DQ, Liu WC (2011). A new hadrosauriform dinosaur from the early cretaceous of Gansu Province, China. Acta Geol. Sin..

[24] Yang JT, You HL, Li DQ, Kong DL (2013). First discovery of polacanthine ankylosaur dinosaur in Asia. Vertebr. Palasiat..

[25] Li N, Li DQ, Peng GZ, You HL (2024). The first stegosaurian dinosaur from Gansu Province, China. Cretac. Res..

[26] Li BX, Xu FX, Ma QH, Pan HZ, Wang SQ, Li ZW (1982). Middle Jurassic Strata of Wangjiashan Basin, Jingyuan, Gansu. J. Stratigr..

[27] Zhang H, He ZL, Jin XL, Zhang H, Li GH, Yang ZY (2009). Sedimentary environments and coal accumulation of the Baojishan-Honghui Basin, eastern Qilian Mountains. Acta Sedmentlolgica Sin..

[28] Du BA (1985). Sporo-pollen assemblages from the Middle Jurassic in the Wangjiashan Basin of Jingyuan, Gansu, and their stratigraphic and paleogeographic significance. Geol. Rev..

[29] Owen R (1842). Report on British fossil reptiles. Rep. Br. Assoc. Adv. Sci..

[30] Seeley HG (1888). The classification of the Dinosauria. Rep. Br. Assoc. Adv. Sci..

[31] Nopcsa F (1915). Die Dinosaurier der Seibenbürgishcen Landisteile Ungarns. Mitteilungen aus dem Jahrbuche der Königlich Ungarischen Geologischen Reichsanstalt.

[32] Marsh OC (1877). A new Order of extinct Reptilia (Stegosauria) from the Jurassic of the Rocky Mountains. Am. J. Sci..

[33] Haubold H (1990). Ein neuer Dinosaurier (Ornithischia, Thyreophora) aus dem unteren Jura des nordlichen Mitteleuropa. Revue de paleobiologie.

[34] Gilmore CW (1914). Osteology of the armoured Dinosauria in the United States National Museum, with special reference to the genus *Stegosaurus*. US Natl. Museum Bull..

[35] Norman DB (2019). *Scelidosaurus harrisonii* from the Early Jurassic of Dorset, England: Cranial anatomy. Zool. J. Linn. Soc.-Lond..

[36] Maidment SCR, Norman DB, Barrett PM, Upchurch P (2008). Systematics and phylogeny of Stegosauria (Dinosauria: Ornithischia). J. Syst. Palaeontol..

[37] Butler RJ, Upchurch P, Norman DB (2008). The phylogeny of the ornithischian dinosaurs. J. Syst. Palaeontol..

[38] Maidment SCR, Wei GB (2006). A review of the Late Jurassic stegosaurs (Dinosauria, Stegosauria) from the People's Republic of China. Geol. Mag..

[39] Sereno PC, Dong ZM (1992). The skull of the basal stegosaur *Huayangosaurus taibaii* and a cladistic diagnosis of Stegosauria. J. Vertebr. Paleontol..

[40] Galton PM (1988). Skull bones and endocranial casts of stegosaurian dinosaur *Kentrosaurus* Hennig, 1915 from Upper Jurassic of Tanzania, East Africa. Geol. Palaeontol..

[41] Berman DS, Mcintosh JS (1986). Description of the lower jaw of *Stegosaurus* (Reptilia, Ornithischia). Ann. Carnegie Mus..

[42] Jia CK, Forster CA, Xu X, Clark J (2007). The first stegosaur (Dinosauria, Ornithischia) from the Upper Jurassic Shishugou Formation of Xinjiang, China. Acta Geol. Sin..

[43] Hao BQ, Zhang QN, Peng GZ, Ye Y, You H (2018). Redescription of *Gigantspinosaurus sichuanensis* (Dinosauria, Stegosauria) from the Late Jurassic of Sichuan, Southwestern China. Acta Geol. Sin..

[44] Dong ZM, Zhou SW, Zhang YH (1983). Dinosaurs from the Jurassic of Sichuan. Palaeontol. Sin..

[45] Raven TJ, Maidment SCR (2018). The systematic position of the enigmatic thyreophoran dinosaur *Paranthodon africanus*, and the use of basal exemplifiers in phylogenetic analysis. PeerJ.

[46] Galton PM, Coombs WP (1981). *Paranthodon africanus* (Broom), a stegosaurian dinosaur from the Lower Cretaceous of South Africa. Geobios-Lyon.

[47] Woodruff C, Trexler D, Maidment SCR (2019). Two new stegosaur specimens from the Upper Jurassic Morrison Formation of Montana, USA. Acta Palaeontol. Pol..

[48] Skutschas PP, Gvozdkova VA, Averianov AO, Lopatin AV, Martin T, Schellhorn R (2021). Wear patterns and dental functioning in an Early Cretaceous stegosaur from Yakutia, Eastern Russia. Plos One.

[49] Maidment SCR, Brassey C, Barrett PM (2015). The Postcranial Skeleton of an Exceptionally Complete Individual of the Plated Dinosaur Stegosaurus stenops (Dinosauria: Thyreophora) from the Upper Jurassic Morrison Formation of Wyoming, USA. Plos One..

[50] Zhou, S. W. *The Middle Jurassic Dinosaurian Fauna from Dashanpu*,* Zigong*,* Sichuan*,* Volume 2: Stegosaurs* 1–55 (Sichuan Scientific and Technological Publishing House, 1984).

[51] Hennig E (1915). *Kentrosaurus aethiopicus*, der Stegosauridae des Tendaguru. Sitzungsberichte der Gesellschaft Naturforschender Freunde zu Berlin.

[52] Irmis R (2007). Axial skeleton ontogeny in the Parasuchia (Archosauria: Pseudosuchia) and its implications for ontogenetic determination in archosaurs. J. Vertebr. Paleontol..

[53] Maidment SCR, Wei GB, Norman DB (2006). Re-description of the postcranial skeleton of the Middle Jurassic stegosaur *Huayangosaurus taibaii*. J. Vertebr. Paleontol..

[54] Norman DB (2020). *Scelidosaurus harrisonii* from the Early Jurassic of Dorset, England: postcranial skeleton. Zool. J. Linn. Soc.-Lond..

[55] Barrett PM, Butler RJ, Mundil R, Scheyer TM, Irmis RB, Sanchez-Villagra MR (2014). A palaeoequatorial ornithischian and new constraints on early dinosaur diversification. Proc. R. Soc. B Biol. Sci..

[56] Baron MG, Norman DB, Barrett PM (2017). Postcranial anatomy of *Lesothosaurus diagnosticus* (Dinosauria: Ornithischia) from the Lower Jurassic of southern Africa: implications for basal ornithischian taxonomy and systematics. Zool. J. Linn. Soc.-Lond..

[57] Breeden BT, Raven TJ, Butler RJ, Rowe TB, Maidment SCR (2021). The anatomy and palaeobiology of the early armoured dinosaur *Scutellosaurus lawleri* (Ornithischia: Thyreophora) from the Kayenta Formation (Lower Jurassic) of Arizona. R. Soc. Open Sci..

[58] Barrett PM, Butler RJ, Yates AM, Baron MG, Choiniere JN (2016). New specimens of the basal ornithischian dinosaur *Lesothosaurus diagnosticus* Galton, 1978 from the Early Jurassic of South Africa. Palaeontol. Afr..

[59] Galton PM (1978). Fabrosauridae, the basal family of ornithischian dinosaurs (Reptilia: Ornithopoda). Paläontol. Z..

[60] Colbert, E. H. *A Primitive ornithischian dinosaur from the Kayenta formation of Arizona* 1–55 (Museum of Northern Arizona Press, 1981).

[61] Rosenbaum JN, Padian K (2000). New material of the basal thyreophoran *Scutellosaurus lawleri* from the Kayenta Formation (Lower Jurassic) of Arizona. Paleobios.

[62] Norman DB, Butler RJ, Maidment SCR (2007). Reconsidering the status and affinities of the ornithischian dinosaur *Tatisaurus oehleri* Simmons, 1965. Zool. J. Linn. Soc.-Lond..

[63] Simmons DJ (1965). The non-therapsid reptiles of the Lufeng Basin, Yunnan, China. Fieldiana Geol..

[64] Raven T, Barrett P, Xu X, Maidment SCR (2019). A reassessment of the purported ankylosaurian dinosaur *Bienosaurus lufengensis* from the Lower Lufeng Formation of Yunnan, People's Republic of China. Acta Palaeontol. Pol..

[65] Dong, Z. M. Primitive armoured dinosaur from the Lufeng Basin, China. In *Mesozoic Vertebrate Life* (eds. Tanke D. H. et al.) 237–242 (Indiana University Press, 2001).

[66] Raven TJ, Maidment SCR (2017). A new phylogeny of Stegosauria (Dinosauria, Ornithischia). Palaeontology.

[67] Goloboff PA, Farris JS, Nixon KC (2008). TNT, a free program for phylogenetic analysis. Cladistics.

[68] Casamiquela RM (1967). Un nuevo dinosaurio ornitischio Triásico (*Pisanosaurus mertii*: Ornithopoda) de la Formación Ischigualasto, Argentina. Ameghiniana.

