# Supplementary material for: A new stegosaur (Dinosauria: Ornithischia) from the Middle Jurassic of Gansu Province, China
Source: Sci Rep. 2024 Jul 2;14:15241. doi: 10.1038/s41598-024-66280-x (PMC11219857; doi:10.1038/s41598-024-66280-x)
Supplement: Supplementary file 1 — Supplementary Information 1. [file 41598_2024_66280_MOESM1_ESM.docx]

**A new stegosaur (Dinosauria: Ornithischia) from the Middle Jurassic of Gansu Province, China**

Li Ning^a,^*, Susannah C. R. Maidment^b^, Li Daqing^c^, You Hailu^d,e^, Peng Guangzhao^f^

^a^ School of Earth Sciences and Resources, China University of Geosciences (Beijing), Beijing, China

^b^ Fossil Reptiles, Amphibians and Birds Section, Natural History Museum, London, U.K.

^c^ Institute of Vertebrate Paleontology, Gansu Agricultural University, Lanzhou, Gansu, China

^d^ Key Laboratory of Vertebrate Evolution and Human Origins, Institute of Vertebrate Paleontology and Paleoanthropology, Chinese Academy of Sciences, Beijing, China

^e^ College of Earth and Planetary Sciences, University of Chinese Academy of Sciences, Beijing, China

^f^ Zigong Dinosaur Museum, Zigong, Sichuan, China

*Corresponding author. Email addresses: liningcq@qq.com.

**Character list**

1. Snout, depth: depth to length ratio of maxilla coded as continuous.
2. Teeth: number coded as meristic.
3. Teeth: Number of denticles on mesial side of maxillary teeth.
4. Premaxilla: Height to length ratio of subnarial portion coded as continuous.
5. Cervical vertebrae: number coded as meristic.
6. Dorsal vertebrae: neural arch to neural canal height ratio as continuous.
7. Dorsal vertebrae: centrum height to neural arch height ratio coded continuously.
8. Dorsal vertebrae: centrum height to neural arch height ratio coded continuously.
9. Dorsal vertebrae: neural spines length (measured at the base) to centrum length ratio coded continuously. (new character)
10. Dorsal vertebrae: number coded as meristic.
11. Scapula: proximal plate area to coracoid area ratio coded continuously.
12. Humerus: ratio of width of distal end to minimum shaft width coded continuously.
13. Humerus: ratio of transverse width of distal end to length coded continuously.
14. Humerus: anterior iliac process length to humerus length coded continuously.
15. Ulna: proximal width to length ratio coded continuously.
16. Ratio of ulna length to humerus length coded continuously.
17. Ratio of radius length to humerus length coded continuously.
18. Metacarpal II to humerus length ratio coded continuously.
19. Ilium: anterior iliac process to acetabular length ratio coded continuously.
20. Ilium: ratio of acetabular length to dorsoventral height of pubic peduncle of ilium coded continuously.
21. Pubis: prepubis to postpubis length ratio coded continuously.
22. Pubis: postpubis to acetabular length ratio coded continuously.
23. Femur: length to humerus length ratio coded continuously.
24. Femur: length to tibia length ratio continuously.
25. Metatarsal IV: ratio of length to width coded as continuous. Maximum length and maximum widths used.
26. Skull, overall shape in posterior view: deeper than wide (0); wider than deep (1).
27. Premaxilla: process projecting caudodorsally from caudolateral corner - gracile (0); robust (1).
28. Premaxilla: Caudodorsally projecting process from craniomedial border extends dorsally to be- visibile on skull roof in dorsal view (0); not visible on skull roof (1).
29. Premaxilla: Broad ‘V’ or ‘U’ shaped notch between premaxillae on the midline absent (0); present (1).
30. Maxilla: tooth row inset medially from the lateral surface of the maxilla absent (0); present (1).
31. Lacrimal: contacts prefrontal (0); doesn’t contact prefrontal (1).
32. Frontals: rostrocaudally longer than wide transversely (0); wider than long (1).
33. Frontals: form the dorsal rim of the orbit (0); supraorbital elements form the dorsal rim of the orbit (1).
34. Parietals, dorsal surface: convex (0); flat (1).
35. Quadrate: fossa/fenestra absent (0); present (1).
36. Quadrate: proximal head strongly transversely compressed, absent (0); present (1)
37. Quadrate: head is strongly arched posteriorly relative to the shaft, absent (0); present (1).
38. Quadrate: axis extending through condyles in posterior view orientated transversely (0); orientated strongly ventromedially (1).
39. Quadrate: contact with paroccipital process unfused (0); fused (1).
40. Quadrate: lateral ramus present (0); absent (1).
41. Quadratojugal: rectangular shape (0); possesses dorsal process that extends to craniolateral surface of quadrate in lateral view (1).
42. Basioccipital: exit for vagus nerve braincase: posterior surface (0); lateral surface (metotic fissure) (1).
43. Jaw joint: ventral to tooth row (0); level with tooth row (1).
44. Dentary: postdentary bones greater in rostrocaudal length than dentary (0); shorter (1).
45. Dentary: tooth row in lateral view visible (0); not visible (1).
46. Dentary: tooth alveoli face dorsally (0); dorsomedially (1).
47. Dentary: tooth row in lateral view straight (0); sinuous (1).
48. Tooth crowns: striations not confluent with denticles (0); confluent with denticles (1).
49. Tooth crowns: asymmetric (0); symmetric (1).
50. Teeth: diastema between predentary facet on the dentary and first tooth present (0); absent (1).
51. Premaxillary teeth: present (0); absent (1).
52. Maxillary teeth: cingulum absent (0); present (1).
53. Skull roof: cortical remodelling absent (0); present (1).
54. Skull roof: cortical remodelling present in only some bones (0); present in all bones, along with the fusion of dermal ossifications, so that the antorbital and supratemporal fenestrae are closed (1).
55. Axis: neural spine triangular in lateral view (0); sub-rectangular in lateral view (1).
56. Axis: ventral margin in lateral view flat (0); concave (1).
57. CV3: centrum ventral margin straight (0); concave upwards (1).
58. Cervical vertebrae: longer anterposteriorly than wide transversely (0); wider than long (1).
59. Posterior cervical vertebrae: postzygapophyses not greatly elongated (0); greatly elongated and project over the back of the posterior centrum facet (1).
60. Anterior dorsal vertebrae: prezygapophyses are separated and face each other dorsally (0); joined ventrally and face dorsomedially (1).
61. Dorsal vertebrae: cranial and caudal articular facets on centra flat to slightly concave (0); strongly convex (1).
62. Dorsal vertebrae: all centra longer than wide (0); wider than long (1).
63. Dorsal vertebrae: transverse processes project approximately horizontally (0); at a high angle to the horizontal (1).
64. Dorsal vertebrae: parapophyses are well developed that held on stalks at the base of the diapophyses; (0); poorly developed (1). (new character)
65. Dorsasacral vertebrae ribs: don’t fuse (0); fuse to dorsal margins of first true sacral vertebrae (1); fuse to medial margin of preacetabular process of ilium (2).
66. Sacral rod vertebrae: keel present (0); absent (1).
67. Anterior caudal vertebrae: dorsal process on transverse process absent (0); present (1).
68. Anterior caudal vertebrae: dorsal process on transverse process proximal to centrum (0); distal to centrum (2).
69. Anterior caudal vertebrae: transverse processes on cd3 posteriorly are directed laterally (0); directed strongly ventrally (1).
70. Anterior caudal vertebrae: neural spine height less than or equal to the height of the centrum (0); greater than the height of the centrum (1).
71. Anterior caudal vertebrae: bulbous swelling at tops of neural spines absent (0); present (1).
72. Caudal vertebrae: prezygapophyses extend craniodorsally (0); extend cranially (1).
73. Caudal vertebrae: postzygapophyses extend cranially over caudal articular facet (0); do not (1).
74. Caudal vertebrae: transverse processes on distal half of tail present (0); absent (1).
75. Caudal vertebrae: neural spines bifurcated (0); not bifurcated (1).
76. Posterior caudal vertebrae: centra are elongate (0); equidimensional (1).
77. Scapula: acromial process in lateral view, convex upwards dorsally (0); quadrilateral with a posterordorsal corner (1).
78. Scapula: acromial process projects dorsally (0); projects laterally (1).
79. Scapula: blade, distally expanded (0); parallel sided (1).
80. Coracoid: sub-circular outline (0); anteroposteriorly longer than dorsoventrally high (1).
81. Coracoid: in lateral view, foramen present (0); notch present (1).
82. Humerus: triceps tubercle and descending ridge posterolateral to the deltopectoral crest absent (0); present (1).
83. Radius: expanded transversely at proximal end (0); not expanded (1).
84. Metacarpals I and V: shorter than metacarpals II, III and IV (0); longer (1).
85. Ungual phalanges: Manual and pedal unguals claw–shaped (0); hoof–shaped (1).
86. Ilium: anterior iliac process lies approximately horizontally (0); strongly angled ventrally (1).
87. Ilium: anterior iliac process projects roughly parallel to the parasagittal plane (0); diverges widely from the parasagittal plane (1).
88. Ilium: horizontal lateral enlargement absent (0); present (1).
89. Ilium: horizontal lateral enlargement incipient (small) (0); large (1).
90. Ilium: supra–acetabular flange projects at 90 degrees from the anterior iliac process absent (0); present (1).
91. Ilium: posterior iliac process, distal shape tapers (0); blunt (1).
92. Ilium: medial processes on posterior iliac processes absent (0); present (1).
93. Ilium: ventromedial flange backing the acetabulum absent (0); present (1).
94. Ilium: preacetabular process has inverted C-shaped cross section that is laterally convex and medially concave (0); does not i.e. transversely compressed (1).
95. Ilio–sacral block: Five or more sacral vertebrae (0); four or fewer sacral vertebrae (1).
96. Ilio–sacral block: Posterior sacral rib angled laterally (0); posterolaterally (1).
97. Ilio–sacral block: dorsal shield of sacrum is perforated by foramina in between ribs (0); is solid with no foramina (1).
98. Ischium: convex proximal margin within the acetabulum absent (0); present (1).
99. Ischium: dorsal surface of shaft is straight (0); has a distinct angle at approximately midlength (1).
100. Ischium: posterior end of ischium, expanded relative to the shaft (0); not expanded and tapers (1).
101. Pubis: obturator notch is backed by posterior pubic process absent (0); present (1).
102. Pubis: acetabular portion faces laterally, posteriorly and dorsally (0); faces wholly laterally (1).
103. Pubis: anterior end of prepubis expanded dorsally absent (0); present (1).
104. Femur: Fourth trochanter prominent and pendant (0); present as a rugose ridge (1); absent (2).
105. Femur: anterior trochanter fusion to greater trochanter in adults - unfused (0); fused (1).
106. Metatarsal V: present (0); absent (1).
107. Pedal digit I: present (0); absent (1).
108. Pedal digit III: has 4 or more phalanges (0); has 3 phalanges (1); has 2 or fewer phalanges (2). Code as ordered.
109. Pedal digit IV: has 5 phalanges (0); has 4 phalanges (1); has 3 or fewer phalanges (2). Code as ordered.
110. Dermal armour: including scutes, and/or spines and/or plates absent (0); present (1).
111. Plates and spines: two parasagittal rows of plates and/or spines absent (0); present (1).
112. Cervical collars: U–shaped cervical collars composed of keeled scutes absent (0); present (1).
113. Osteoderms: mosaic of small osteoderms between larger osteoderms on the ventral surfaces of the neck, trunk, and proximal portions of the limbs absent (0); present (1).
114. Parascapular spine: absent (0); present (1).
115. Dorsal plates: have a thick central portion like a modified spine (0); have a generally transversely thin structure, except at the base (1).
116. Parasagittal rows of dermal armour: paired (0); alternating either side of the midline (1).
117. Ossified epaxial tendons: present (0); absent (1).

**Scores of new characters and source of scorings**

Except *Baiyinosaurus*, the information of these scores from the photos of previously published literature.

| Taxa | Scores of character 9 | Scores of character 64 | Sources of Scorings |
| --- | --- | --- | --- |
| *Lesothosaurus* | 1 | 0 | Baron *et al.* (2017) |
| *Scutellosaurus* | 0.72 | 0 | Breeden *et al.* (2021) |
| *Emausaurus* | ? | ? | / |
| *Scelidosaurus* | 0.81 | 0 | Norman (2020) |
| *Huayangosaurus* | 0.55 | 1 | Maidment *et al.* (2006); Zhou (1984) |
| *Dacentrurus* | 0.58 | 1 | Galton (1991) |
| *Miragaia* | ? | ? | / |
| *Loricatosaurus* | 0.59 | 1 | Galton (1985) |
| *Kentrosaurus* | 0.72 | 1 | Galton (1982); Hennig (1915) |
| *Paranthodon* | ? | ? | / |
| *Chungkingosaurus* | ? | 1 | Maidment and Wei (2006); Dong *et al.* (1983) |
| *Tuojiangosaurus* | 0.56 | 1 | Dong *et al.* (1977) |
| *Gigantspinosaurus* | 0.74 | 1 | Hao *et al.* (2018) |
| *Stegosaurus homheni* | ? | 1 | Maidment *et al.* (2008) |
| *Stegosaurus stenops* | 0.5 | 1 | Maidment *et al.* (2015) |
| *Hesperosaurus* | 0.6 | 1 | Carpenter *et al.* (2001) |
| *Gastonia* | ? | 1 | Kinneer *et al.* (2016) |
| *Sauropelta* | 0.78 | ? | Ostrom (1970) |
| *Euoplocephalus* | ? | ? | / |
| *Jiangjunosaurus* | ? | ? | / |
| *Laquintasaura* | 0.74 | ? | Barrett *et al.* (2014) |
| *Alcovasaurus* | ? | 1 | Galton and Carpenter (2016) |
| *Pisanosaurus* | ? | ? | / |
| *Adratiklit* | ? | 1 | Maidment *et al.* (2020) |
| *Isaberrysaura* | ? | ? | / |
| *Bashanosaurus* | 0.56 | 0 | Dai *et al.* (2022) |
| *Baiyinosaurus* | 0.75 | 0 | This study |

**References**

1. Baron, M. G., Norman, D. B. & Barrett, P. M. Postcranial anatomy of *Lesothosaurus diagnosticus* (Dinosauria: Ornithischia) from the Lower Jurassic of southern Africa: implications for basal ornithischian taxonomy and systematics. *Zool. J. Linn. Soc.-Lond.* **179**, 125-168 (2017).

2. Breeden, B. T., Raven, T. J., Butler, R. J., Rowe, T. B. & Maidment, S. C. R. The anatomy and palaeobiology of the early armoured dinosaur *Scutellosaurus lawleri* (Ornithischia: Thyreophora) from the Kayenta Formation (Lower Jurassic) of Arizona. *Royal Society Open Science*. **8**, 201676 (2021).

3. Norman, D. B. *Scelidosaurus harrisonii* from the Early Jurassic of Dorset, England: postcranial skeleton. *Zool. J. Linn. Soc.-Lond.* **189**, 47-157 (2020).

4. Maidment, S. C. R., Wei, G. B. & Norman, D. B. Re-description of the postcranial skeleton of the Middle Jurassic stegosaur *Huayangosaurus taibaii*. *J. Vertebr. Paleontol.* **26**, 944-956 (2006).

5. Zhou, S. W. *The Middle Jurassic Dinosaurian Fauna from Dashanpu, Zigong, Sichuan, Volume 2: Stegosaurs* 1-55 (Sichuan Scientific and Technological Publishing House, 1984).

6. Galton, P. M. Postcranial remains of stegosaurian dinosaur *Dacentrurus* from Upper Jurassic of France and Portugal. *Geologica et Palaeontologica*. **25**, 299-327 (1991).

7. Galton, P. M. British plated dinosaurs (Ornithischia, Stegosauridae). *J. Vertebr. Paleontol.* **5**, 211-254 (1985).

8. Galton, P. M. The postcranial anatomy of stegosaurian dinosaur *Kentrosaurus* from the Upper Jurassic of Tanzania, East Africa. *Geologica et Palaeontologica*. **15**, 139-160 (1982).

9. Hennig, E. *Kentrosaurus aethiopicus*, der Stegosauridae des Tendaguru. *Sitzungsberichte der Gesellschaft Naturforschender Freunde zu Berlin*. **1915**, 219-247 (1915).

10. Dong, Z. M., Zhou, S. W. & Zhang, Y. H. Dinosaurs from the Jurassic of Sichuan. *Palaeontologica Sinica*. **162**, 1-151 (1983).

11. Dong, Z. M., Li, X. & Zhou, S. W. On the stegosaurian remain from Zigong (Tzekung), Szechuan Province. *Vertebrata Palasiatica*. **15**, 312 (1977).

12. Hao, B. Q., Zhang, Q. N., Peng, G. Z., Ye, Y. & You, H. Redescription of *Gigantspinosaurus sichuanensis* (Dinosauria, Stegosauria) from the Late Jurassic of Sichuan, Southwestern China. *Acta geologica Sinica (English Edition)*. **92**, 431-441 (2018).

13. Maidment, S. C. R., Norman, D. B., Barrett, P. M. & Upchurch, P. Systematics and phylogeny of Stegosauria (Dinosauria: Ornithischia). *J. Syst. Palaeontol.* **6**, 367-407 (2008).

14. Maidment, S. C. R., Brassey, C. & Barrett, P. M. The Postcranial Skeleton of an Exceptionally Complete Individual of the Plated Dinosaur *Stegosaurus stenops* (Dinosauria: Thyreophora) from the Upper Jurassic Morrison Formation of Wyoming, U.S.A. *Plos One*. **10**, e138352 (2015).

15. Carpenter, K., Miles, C. A. & Cloward, K. New primitive stegosaur from the Morrison Formation, Wyoming. In *The Armored Dinosaur* (ed. Carpenter K.) 55-76 (Indiana University Press, 2001).

16. Kinneer, B., Carpenter, K. & Shaw, A. Redescription of Gastonia burgei (Dinosauria: Ankylosauria, Polacanthidae), and description of a new species. *Neues Jahrbuch für Geologie und Paläontologie - Abhandlungen*. **282**, 37-80 (2016).

17. Ostrom, J. H. Stratigraphy and paleontology of the Cloverly Formation (Lower Cretaceous) of the Bighorn Basin area, Wyoming and Montana. *B. Peabody Mus. Nat. Hi.* (1970).

18. Barrett, P. M., Butler, R. J., Mundil, R., Scheyer, T. M., Irmis, R. B. & Sanchez-Villagra, M. R. A palaeoequatorial ornithischian and new constraints on early dinosaur diversification. *Proceedings of the Royal Society B: Biological Sciences*. **281**, 20141147 (2014).

19. Galton, P. M. & Carpenter, K. The plated dinosaur *Stegosaurus longispinus* Gilmore, 1914 (Dinosauria: Ornithischia; Upper Jurassic, western USA), type species of *Alcovasaurus* n. gen. *Neues Jahrbuch für Geologie und Paläontologie - Abhandlungen*. **279**, 185-208 (2016).

20. Maidment, S. C. R., Raven, T. J., Ouarhache, D. & Barrett, P. M. North Africa's first stegosaur: Implications for Gondwanan thyreophoran dinosaur diversity. *Gondwana Res.* **77**, 82-97 (2020).

21. Dai, H., Li, N., Maidment, S. C. R., Wei, G. B., Zhou, Y. X. & Hu, X. F. et al. New Stegosaurs from the Middle Jurassic Lower Member of the Shaximiao Formation of Chongqing, China. *J. Vertebr. Paleontol.* **41**, (2022).
